# Supplementary material for: The genome sequence of a ground beetle, Ophonus ardosiacus (Lutshnik, 1922)
Source: Wellcome Open Res. 2023 Aug 17;8:353. [Version 1] doi: 10.12688/wellcomeopenres.19849.1 (PMC11259774; doi:10.12688/wellcomeopenres.19849.1)
Supplement: Peer review history

# Reviewer report: Reviewer response for version 1

**DOI:** 10.21956/wellcomeopenres.21982.r87651

**Published:** 2024-07-19

**Authors:** Ping Wang

**Affiliations:** 1 Yangtze University, Jingzhou,, China

Dear Authors,

 I have put forward some views that are only my own in the following sections respectively, thank you.

- This is an interesting manuscript that provides information on the genome sequencing of *Ophonus ardosiacus* . The manuscript presents a comprehensive genome assembly of *Ophonus ardosiacus* , a ground beetle, as part of the Darwin Tree of Life Project. The assembly appears to be of high quality, with a significant portion of the genome scaffolded into chromosomal pseudomolecules and a detailed annotation of protein-coding genes. The use of advanced sequencing technologies and the integration of Hi-C data for scaffolding are commendable.
- The background information on *O. ardosiacus* is informative, but it could be enhanced by discussing the ecological and evolutionary significance of ground beetles in general, and how this specific species contributes to the ecosystem. This would provide a broader context for the importance of the genome sequence.
- The authors have provided detailed information on the sequencing and assembly methods, which is excellent. However, it would be beneficial to include a brief explanation of the technologies (e.g., Pacific Biosciences HiFi, 10X Genomics) for readers who may not be familiar with these terms.
- The BUSCO scores indicate a high completeness of the assembly, which is commendable. It would be helpful to briefly discuss any potential limitations or areas for improvement in the assembly process.
- The annotation of 40,995 protein-coding genes is a significant achievement. It would be beneficial to provide insights into the functional diversity of these genes, perhaps by comparing them to other related species or discussing potential novel genes that could be of interest.
- The manuscript mentions the potential use of the genome sequence in studies assessing invertebrate communities for agricultural areas. It would be helpful to expand on this, discussing specific research questions or applications that could benefit from this resource.
- The conclusion could be strengthened by summarizing the key contributions of this genome assembly to the field of entomology and ecology, and how it fits into the broader goals of the Darwin Tree of Life Project.
- The manuscript is well-prepared and presents a valuable resource for the scientific community. With the suggested improvements, I believe it would be a strong contribution to Wellcome Open Research.

Are sufficient details of methods and materials provided to allow replication by others?

Yes

Is the rationale for creating the dataset(s) clearly described?

Yes

Are the datasets clearly presented in a useable and accessible format?

Yes

Are the protocols appropriate and is the work technically sound?

Yes

Reviewer Expertise:

Taxonomy; Entomology

I confirm that I have read this submission and believe that I have an appropriate level of expertise to confirm that it is of an acceptable scientific standard.

# Reviewer report: Reviewer response for version 1

**DOI:** 10.21956/wellcomeopenres.21982.r65599

**Published:** 2023-09-14

**Authors:** Yi-Ming Weng

**Affiliations:** 1 Department of Entomology, University of Wisconsin Madison, Madison, WI, USA

This paper reports a very complete genome assembly for a carabid beetle, *Ophonus ardosiacus*. The number of coding gene was quite high comparing to many other ground beetle species, despite of the fact that only protein was used to train the model (usually more genes with RNA data to jointly train the gene model with proteins). I wonder which protein database was used to in modeling the gene in the braker2 pipeline.

Are sufficient details of methods and materials provided to allow replication by others?

Partly

Is the rationale for creating the dataset(s) clearly described?

Yes

Are the datasets clearly presented in a useable and accessible format?

Yes

Are the protocols appropriate and is the work technically sound?

Yes

Reviewer Expertise:

Population genetics, evolutionary genomics, taxonomy of Carabidae.

I confirm that I have read this submission and believe that I have an appropriate level of expertise to confirm that it is of an acceptable scientific standard.
